# In Vivo Detection of Activated Platelets Allows Characterizing Rupture of Atherosclerotic Plaques with Molecular Magnetic Resonance Imaging in Mice

**DOI:** 10.1371/journal.pone.0045008

**Published:** 2012-09-13

**Authors:** Dominik von Elverfeldt, Constantin von zur Muhlen, Kristina Wiens, Irene Neudorfer, Andreas Zirlik, Mirko Meissner, Peg Tilly, Anne-Laure Charles, Christoph Bode, Karlheinz Peter, Jean-Etienne Fabre

**Affiliations:** 1 Department of Radiology/Medical Physics, University Hospital, Freiburg, Germany; 2 Department of Cardiology and Angiology I, University Heart Center, Freiburg, Germany; 3 Institut de Génétique et de Biologie Moléculaire et Cellulaire (IGBMC), Institut National de Santé et de Recherche Médicale (INSERM) U964/Centre National de Recherche Scientifique (CNRS) UMR 7104/Université de Strasbourg, Illkirch, France; 4 Baker IDI Heart & Diabetes Institute, Melbourne, Australia; University Medical Center Utrecht, The Netherlands

## Abstract

**Background:**

Early and non-invasive detection of platelets on micro atherothrombosis provides a means to identify unstable plaque and thereby allowing prophylactic treatment towards prevention of stroke or myocardial infarction. Molecular magnetic resonance imaging (mMRI) of activated platelets as early markers of plaque rupture using targeted contrast agents is a promising strategy. In this study, we aim to specifically image activated platelets in murine atherothrombosis by *in vivo* mMRI, using a dedicated animal model of plaque rupture.

**Methods:**

An antibody targeting ligand-induced binding sites (LIBS) on the glycoprotein IIb/IIIa-receptor of activated platelets was conjugated to microparticles of iron oxide (MPIO) to form the LIBS-MPIO contrast agent causing a signal-extinction in T2*-weighted MRI. *ApoE^−/−^* mice (60 weeks-old) were fed a high fat diet for 5 weeks. Using a small needle, the surface of their carotid plaques was scratched under blood flow to induce atherothrombosis. *In vivo* 9.4 Tesla MRI was performed before and repetitively after intravenous injection of either LIBS-MPIO versus non-targeted-MPIO.

**Results:**

LIBS-MPIO injected animals showed a significant signal extinction (p<0.05) in MRI, corresponding to the site of plaque rupture and atherothrombosis in histology. The signal attenuation was effective for atherothrombosis occupying ≥2% of the vascular lumen. Histology further confirmed significant binding of LIBS-MPIO compared to control-MPIO on the thrombus developing on the surface of ruptured plaques (p<0.01).

**Conclusion:**

*in vivo* mMRI detected activated platelets on mechanically ruptured atherosclerotic plaques in ApoE^−/−^ mice with a high sensititvity. This imaging technology represents a unique opportunity for noninvasive detection of atherothrombosis and the identification of unstable atherosclerotic plaques with the ultimate promise to prevent strokes and myocardial infarctions.

## Introduction

Cardiovascular diseases are still a major factor of mortality and morbidity [1] in developed countries and are emerging as a major health care problem in developing countries. Early detection of atherothrombosis would improve the management of acute coronary diseases.

The majority of fatal myocardial infarctions are caused by disruption of the fibrous cap that covers atherosclerotic plaques. Subsequent exposure of molecules such as von Willebrandt Factor and collagen mediate platelet adhesion and activation, leading to thrombosis. Depending on local factors, such as the size of the rupture or the inflammatory status of the plaque [2], thrombosis is limited or conversely becomes obstructive. It is therefore highly desirable to detect early non-obstructive thrombosis before it induces myocardial infarction or stroke. Indeed timely angioplasty is beneficial in non-ST elevation acute coronary syndrome [3], and identifying ruptured lesions, which have not yet resulted in occlusive disease, could help in patient management.

Contrary to conventional angiography [4], PET-CT imaging with fluordeoxyglucose allows detection of inflammatory processes in rupture-prone atherosclerotic plaques [5]. However, this approach is limited by a poor spatial resolution and does not indicate whether the plaque has already ruptured and initiated limited thrombosis. In this context, MRI with specific contrast agents [6] is of high interest. Indeed, MRI characterizes plaques [7] and molecular contrast agents can target cellular receptors activated by atherothrombosis [8]. One molecular determinant of activated platelets is the highly abundant and uniquely specific integrin α_IIb_ß_3_. We recently described a MRI contrast agent targeting ligand-induced binding sites (LIBS) of the activated integrin α_IIb_ß_3_ [9], [10], [11], [12], which thereby targets specifically activated platelets.

The efficiency of this platelet-targeting strategy to image activated platelets has been demonstrated in a proof of concept approach using an *in vivo* model of ferric chloride-induced thrombosis in the carotid artery of mice [9]. However, it is not known whether this strategy can detect atherothrombosis, and whether sensitivity would be sufficient.

We are now providing data demonstrating that this platelet targeting approach can be used in magnetic resonance imaging to identify small thrombi occurring on *in vivo* ruptured atherosclerotic plaques. This technology provides a unique perspective towards the imaging of unstable atherosclerotic plaques.

## Methods

### Construction of the MRI Contrast Agent Specific for Activated Platelets

The monoclonal antibody (mAb) anti-LIBS 145 binds to α_IIb_ß_3_ only in its active conformation [13]. Cloning, generation and production of the single-chain antibody anti-LIBS 145 has been described in detail elsewhere [14]. The generation and purification of the irrelevant control single-chain antibody was also described elsewhere [15].

The contrast agent was constructed using autofluorescent cobalt-functionalised MPIOs (sized 1 µm, Dynal Biotech, Oslo, Norway) conjugated with the histidine-tag of the LIBS/control single-chain antibody [9], [11]. A magnet was used to separate unbound from MPIO-bound antibody.

### Animal Model

Our studies are in compliance with the position of American Heart Association on research animal use. This study followed the national guidelines, and was approved by the institutional animal care committee of the University of Freiburg, Germany (permit No. 35/9185.81/G-09/47).

*ApoE^−/−^* mice aged of 60 weeks and fed a high fat diet for 5 weeks were anesthetized with isoflurane. A venous catheter with a 120 cm long tube (Portex, Smiths Medical International, USA) was placed in the tail vein. A 30-gauge needle with its bended tip was introduced through a branch in the external carotid and advanced up to a plaque. The tip was applied to the plaque to scratch its surface [2]. The collateral artery was ligated, the wound sutured and 8 units of heparin were injected to prevent vessel occlusion.

### In Vivo Experimental and MRI Protocol of Contrast Agent Injection

Mice were consecutively and randomly assigned to either LIBS-MPIO or control-MPIO group before performing the MRI. The mice were placed in the custom made animal bed, connected to an ECG and breathing-rate monitor, and transferred to the MRI system, a 94/20 Bruker BioSpec, Bruker, Germany.

The body temperature and breathing rate were continuously monitored. MRI was performed employing a quadrature whole body birdcage resonator (35 mm inner diameter) and the following imaging protocol.

After an iterative 1^st^ and 2^nd^ order shimming routine the geometry of a coronal, ECG triggered 2D-FLASH (TE/TR: 4.4 ms/250 ms) with 15 slices was adjusted on a standard FLASH based multi slice pilot scan to be in plane with the course of the common carotid arteries. Perpendicular to these images a 3D-FLASH (Matrix: 256×256×64) with TE/TR: 2.8 ms/20 ms, a flip angle of 25°, a bandwidth of 55 kHz, 2 averages, and a field of view of 27×27×9 mm^3^ resulting in a voxel resolution of 105×105×141 µm^3^ and a total scan time of 8 min 11 s was run. This sequence was optimised to achieve a highly hyperintense blood signal from the carotid arteries. The injection was performed only when the hyperintense blood signal was identified.

### Quantification of MPIO-induced Signal Void

Image evaluation was Matlab® based, as described elsewhere (9). In brief, we calculated for each time point the quotient of mean signal intensity of the ROI in the vessel with plaque-rupture divided by the mean of the ROI in the contralateral, non-injured vessel. These quotients were then normalized on the baseline 3D FLASH starting value, gaining a time series of the fractional amount of remaining signal after contrast agent injection.

### Tissue Harvesting and Histology

After performing in vivo MRI, animals were euthanized and perfused throught the left ventricle with saline. The injured and contralateral carotid artery were embedded in OCT TissueTec (Sakura Finetec, Netherlands) and frozen for histology. Thrombosis was quantified through immunodetection of platelets using rat anti-mouse glycoprotein IIb (CD41) polyclonal antibody (Clone MWReg30, GeneTex, USA), followed by a rabbit anti-rat biotinylated secondary antibody (Vectastain ABC-AP and VectorRed, Vector, Germany). On nine representative sections covering the scratched area of the plaque, thrombus size was quantified in percent of the total vessel lumen using Axiovision Software (Carl Zeiss, Germany). The same sections were used to count the number of MPIOs and the average per section, covering the volume of the superimposed thrombus was calculated. Furthermore, plaque size was measured in percent of total vessel lumen in the same sections, and averaged for each animal. One subset of sections was also stained using a standard Masson-Trichrome protocol.

### Statistics

The in vivo-protocol was performed in 20 mice, 16 of which received LIBS-MPIO and 4 received control-MPIO as contrast agent. The complexity of our animal model led to exclude 9 LIBS-MPIO animals from the statistical analysis. In 7 animals, the exclusion was decided on the baseline scan. In 5 cases the induced thrombus had completely occluded the vessel. In 2 cases a signal void in the contra lateral vessel was observed, and histology indeed showed local occlusion. Finally one animal died during contrast agent injection and for one animal the histological data where lost due to significant problems processing the tissue.

Statistics tests were finally performed on 11 mice (n = 7 for LIBS-MPIO; n = 4 for control-MPIO), using a standardized t-test (MRI signal attenuation and histological MPIO counts) or Pearson’s test (correlation analyses). Differences were considered significant when p<0.05, as calculated with Microsoft Excel™.

## Results

### Mechanical Plaque Rupture and Atherothrombosis

**Figure 1:**
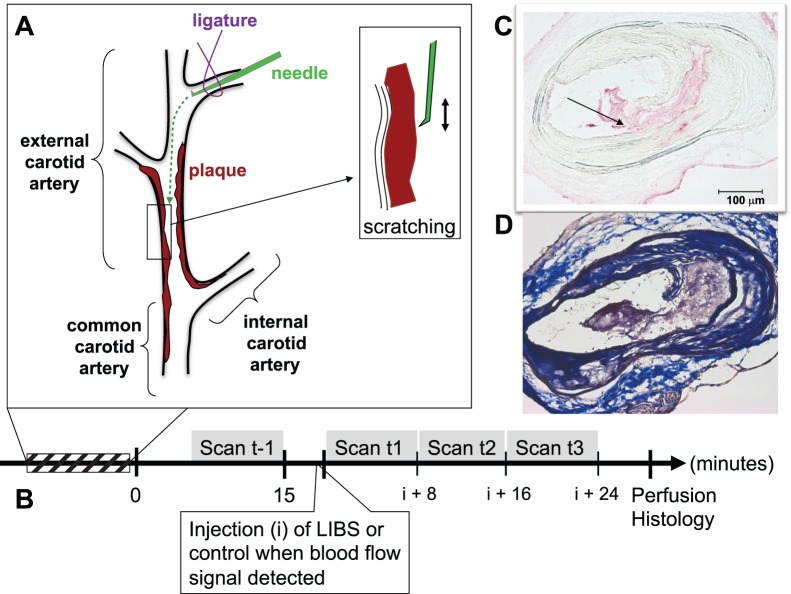
The experimental design. (A) Cartoon depicting the model used for inducing atherothrombosis. A tiny curved needle introduced through a branch of the external carotid was advanced up to the plaque surface under persistent blood flow. After positioning the tip of the needle in front of the plaque, it was applied to the plaque and moved forth and back twice in order to scratch the fibrous cap. (B) The experimental timeline. “I” stands for injection. (C) Transversal histological section of the carotid artery after the scratch injury. Immunohistochemistry depicts platelets and therefore thrombus in red. The scratch with superimposed thrombosis is clearly visible and induced here a small dissection (arrow). A corresponding section of the site of the plaque rupture stained with Masson-Trichrome is depicted in (D).

In all included animals (n = 11), plaques were successfully scratched (Fig. 1). Histology performed in these animals assessed the presence of thrombosis (Fig. 1C). In average, the thrombus occupied 4.83±2.73% of the lumen in the group of mice that received LIBS-MPIO (n = 7) and 3.08±0.02% in the control animals receiving control-MPIO (n = 4).

### Injection of LIBS-MPIO Reduced the MR Signal Only on the Ruptured Side

**Figure 2:**
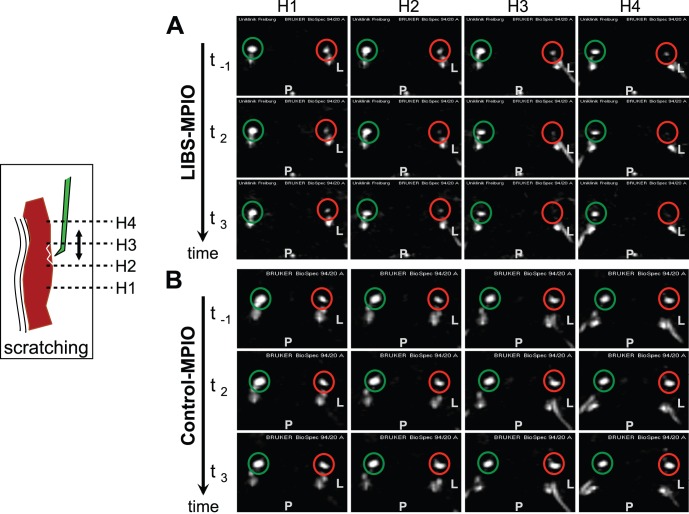
Axial MRI images of the external carotid arteries from two representative animals. Depicted are four consecutive slices (H1-H4) at three time points: t_(−1)_ (before injection), t_2_ (16 minutes after injection) and t_3_ (24 minutes after injection). Red circles indicate the left external carotid artery which underwent surgery and scratching of the plaque, and green circles mark the contra lateral vessel. The animals orientation is indicated by labeling its posterior (P) and left (L) direction. Signal attenuation can be observed in the animal A which received LIBS-MPIO contrast agent whereas the blood signal remains hyperintense in animal B receiving the control-MPIO.

In all scratched and included animals, the white MR signal (red circle) induced by flowing blood in transversal sections of the carotid artery is clearly visible before the injection (Fig. 2, time points t_(−1)_). After the injection, LIBS-MPIOs attenuated the signal in the injured external carotid artery (Fig. 2A, time points t_2_ and t_3_). This is well visible in the second, third and fourth transversal section/column, which corresponds to the developing thrombus in histology. No signal extinction is seen in the non-injured internal carotid artery (green circle). In animals receiving control-MPIO (Fig. 2B), the same procedure failed to attenuate the signal, suggesting that the non-specific contrast agent did not bind at the site of plaque ruptures.

These examples indicate that only LIBS-MPIOs extinguished the MR signal and only on the injury side, which strongly suggest that LIBS-MPIOs recognized activated platelets in atherothrombosis.

### The Threshold for Detecting Ruptured Plaques with Superimposed Thrombus by LIBS-MPIO is 2% of the Vessel Lumen

Since the induced thrombus showed large variations in size due to the intrinsic properties of plaques, we analysed the animals in two sub-groups defined by their thrombus size above or below 2% of the total vessel lumen. The averaged thrombus size in the group of animals with thrombosis occupying more than 2% of the artery lumen was 8.46%±2.93%, while thrombosis occupied only 1.2%±0.22% in the other group.

**Figure 3:**
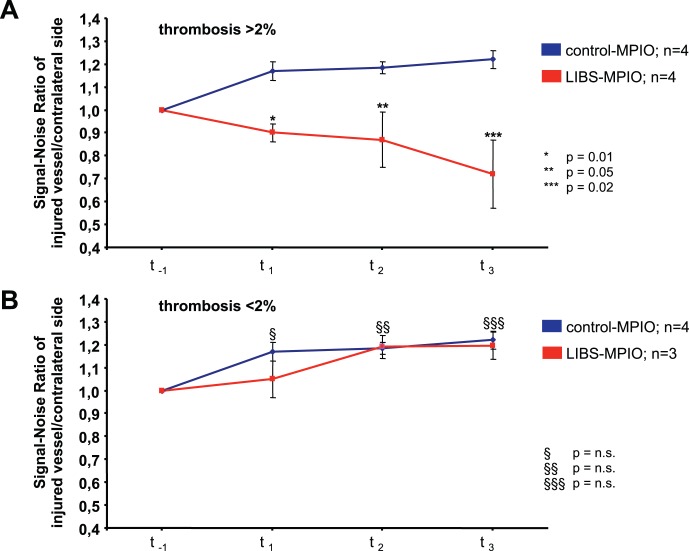
Quantitative analysis of MRI data. Subgroup analysis of animals with thrombus size >2% is shown in Fig. 3A for animals with LIBS-MPIO and control-MPIO injection. At each time point after the injection of LIBS-MPIO, the MR signal detected at the level of the scratched plaque was significantly lower than the signal detected at the contralateral and non-injured plaque level (A, red line). Conversely, the ratio of signals slightly increased after the injection of control-MPIO (A, blue line). In animals with thrombus size <2%, the signal ratio had not been altered by the injection of LIBS-MPIO in comparison to the injection of control-MPIO (3B).

The subgroup analysis of animals with thrombus size comprised between 21% and 2% of the carotid lumen is shown in Fig. 3A for animals with LIBS-MPIO and control-MPIO injection. At each time point after the injection of LIBS-MPIO, the MR signal detected at the level of the scratched plaque was lower than the signal detected at the level of the contralateral and non-injured plaque (Fig. 3A, red line). Conversely, the ratio of signals increased after the injection of control-MPIO (Fig. 3A, blue line).

In animals in which the thrombus had been limited to 2% of the lumen, the signal ratio had not been altered by the injection of LIBS-MPIO, as compared to the injection of control-MPIO (Fig. 3B).

Therefore, we can conclude that LIBS-MPIO attenuated the MR signal specifically on the injured side and that 2% thrombus size in relation to total vessel lumen appears to be the threshold for reliable detection in this model.

### MPIOs were Located on the Injured Side

**Figure 4:**
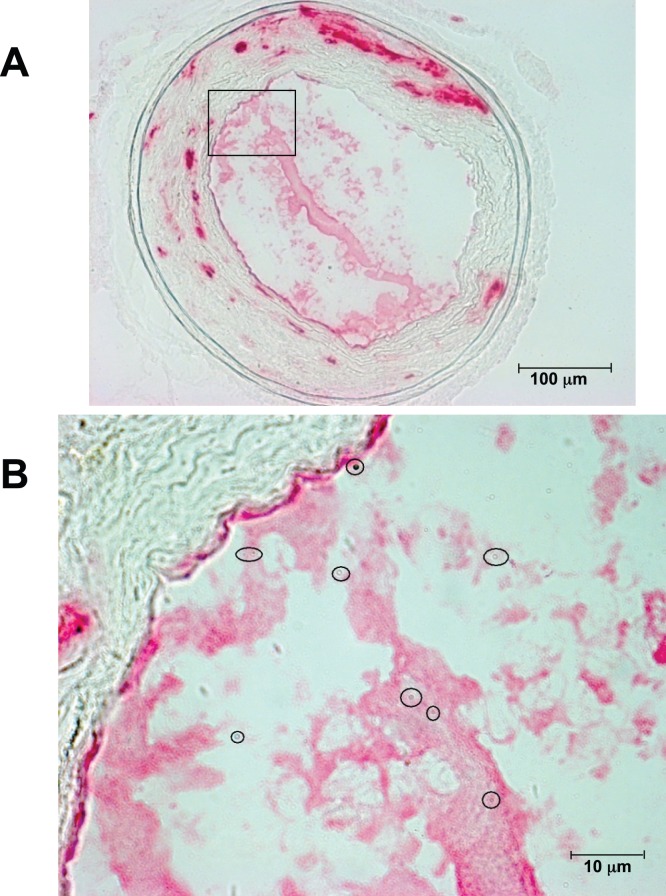
Histological identification of MPIOs. (A): Representative anti-CD41 immunostaining of a carotid artery section from a mouse receiving LIBS-MPIOs. (B) Enlargement of the area from (A) shows MPIOs inside the thrombus (circles). MPIOs can be recognized as regular round structures with a blue halo and a brownish inner colour.

**Figure 5:**
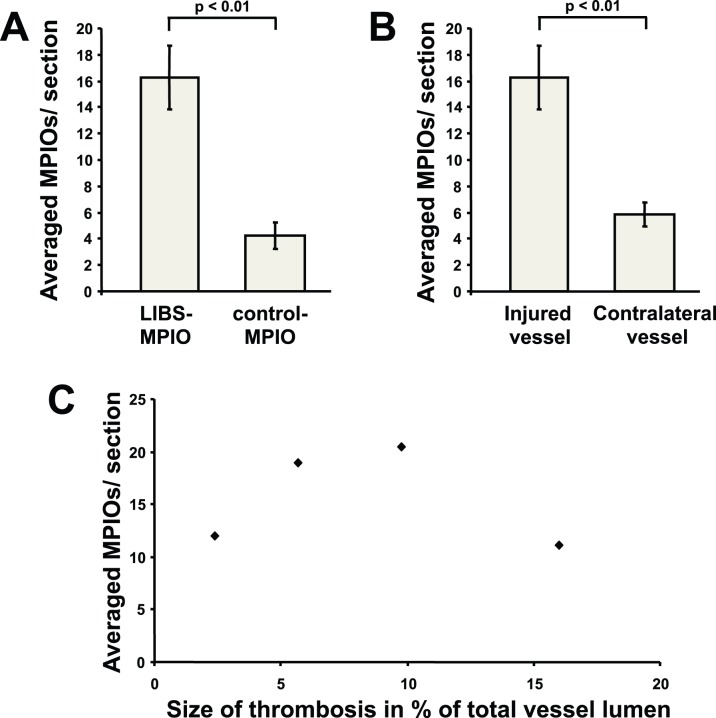
Quantification of MPIOs on histological sections. MPIOs were about four-fold more abundant in animals with LIBS-MPIO-injection as compared to the animals receiving control-MPIO. A similar difference was observed between injured and non injured vessels in animals receiving LIBS-MPIO. Correlation of thrombus size with the number of bound MPIOs in animals with LIBS-MPIO injection is not significant; however the number of MPIOs is in average within a similar range of 10–20 MPIOs per section as expected from the observed signal attenuation.

MPIOs could be identified on histological slides, as shown on Fig. 4. We counted and averaged the number of MPIOs found in 5 histological sections representing the whole thrombus volume in each animal. As shown in Fig. 5A, MPIOs were about four-fold more abundant in slices from animals receiving LIBS-MPIO as compared to the animals receiving control-MPIO (16.25±2.4 vs. 4.25±1.0 per section, p<0.01).

A similar difference was observed between injured and non injured carotids in animals receiving LIBS-MPIO (Fig. 5B: 16.25±2.4 vs. 5.90±1.0 per section, p<0.01).

We calculated the number of MPIOs needed to attenuate the signal. Given a relaxivity r_2_* of 494 L.mmol^−1^.s^−1^, an echo time of 2.8 ms, a vessel diameter of 420 µm, a thrombus occupying 15% of the lumen, and a histological slice thickness of 10 µm, 11.3 MPIOs in average are needed to decrease the signal by 30%. Based on this calculation, the 16.25 MPIOs in average found on histological slices explain well the signal attenuation seen on MRI acquisitions.

Altogether, our data show that injected LIBS-MPIOs recognized specifically fresh atherothrombosis, attenuated the MR signal and thereby can be used to detect even moderate atherothrombosis provided that it occupies more than 2% of the vascular lumen.

## Discussion

Our present results show that atherothrombosis triggered by the mechanical rupture of atherosclerotic plaques was specifically detected by LIBS-MPIO, as assessed by the significant higher number of MPIO on histological slices, which diminished the MR signal through the paramagnetic property of iron oxide.

In previous studies, the injection of LIBS-MPIO in mice decreased the blood flow MRI signal in carotid arteries where thrombosis had been induced by ferric chloride, while a subsequent thrombolytic treatment restored the signal [9]. Because thrombosis in this model is associated with a substantial thrombus load and is stable, it was necessary to test whether this strategy can detect atherothrombosis in vulnerable plaques, in which thrombosis might be limited and labile.

We used 60-weeks old ApoE−/− mice in which plaques developed slowly under chow-fed, allowing the formation of fibrous cap. We then subjected these mice to high fat diet for 5 weeks in order to exacerbate inflammation in the plaque [16] and thereby the plaque thrombogenicity [2]. To induce atherothrombosis, we scratched carotid plaques, because the limited resulting injury is well-suited to test the hypothesis that nascent thrombosis can be detected in vivo by MRI using the LIBS strategy. The LIBS-antibody specifically detects the activated glycoprotein IIb/IIIa-receptor on the surface of activated platelets, and thereby detects developing thrombi. This method is highly sensitive, because the large sizes of the microparticles combined with a high payload of iron allow just a few single MPIO to alter the MRI signal [17], [18].

We observed that the MR signal was diminished following the LIBS-MPIO injection, while histology showed that atherothrombosis had been induced by the scratch. By contrast, we should not observe any change in the ratio of injured/non injured carotid signals in mice receiving control-MPIO. In fact, the ratio increased (Fig. 3, blue lines). This effect can be explained by fibrinolysis of the thrombus that ultimately detached from the vessel wall under the pressure of blood flow. As a consequence the flow increased and augmented the MR-signal. This effect occurred also in animals that had received LIBS-MPIO; however, the MPIOs trapped in the remaining thrombus were sufficient to cause a significant signal decrease.

The paramagnetic effect of iron was linked to the presence of iron oxide particles in atherothrombosis, as shown by histology. We were not surprised to observe some baseline uptake after control-MPIO injection (Fig. 5A), because forming atherothrombosis can non-specifically entrap some of these particles. However, their number was not sufficient to cause a relevant signal loss. In addition, we observed some particles on the surface of the non-injured contralateral atherosclerotic plaques (Fig. 5B), also unable to extinct the MRI signal. The histological analysis confirmed the absence of relevant atherothrombosis (>2%) on the uninjured sides; it is however possible that these particles have been entrapped in minor platelet aggregations occurring at the surface of inflamed and unstable plaques, especially after the 5-weeks of high fat diet [19], [20].

Although the circulation-time for large MPIOs is very short and they are taken up rapidly by the liver and the spleen, MPIOs are even present in significant numbers after 72 min total circulation time (unpublished data). Particles can therefore bind over a long period of time, which explains that we even observe an increase in signal intensity as demonstrated in figure 3. As long as the particles have not bound and accumulated at the site of the thrombus, their contrast effects only average to a slight shortening of T2* of the blood signal. In the images, this effect is negligible due to the sequence-related strong enhancement of inflowing blood as seen in the contralateral carotid artery and in the images using control-MPIO. However, signal evaluation still accounts for this effect by normalizing signal intensities towards the contralateral blood signal.

Thus, we concluded that LIBS-MPIO is a suitable approach for detecting early and small atherothrombosis in mice. The low threshold of detection (2% of the vascular lumen) suggests that the LIBS-MPIO strategy should be efficient at detecting vulnerable/unstable atherosclerotic plaques.

Other approaches are currently under investigation for detecting vulnerable plaques. For example, a radionuclide-based strategy *ex vivo* detected the glycoprotein VI exposed by endothelium injury in carotid arteries [21]. A non contrast-enhanced T1-weighted MRI of methemoglobin detected intracoronary thrombi in patients [22]. However, methemoglobin is also present in plaque hemorrhages and may be the source of false positive signals. MRI contrast agents targeting activated platelets have the theoretical advantage to be more specific. A fibrin-targeted peptide [23], [24] and a cyclic RGD peptide coupled to Gadolinium [25] are promising alternativestrategies for imaging thrombosis. However, our present study is the first to show efficiency at detecting specifically atherothrombosis directly *in vivo*.

Gawaz et al. reported the efficiency of soluble GPVI to identify arterial lesions induced by a wire in ApoE−/− mice [26] or by ex vivo ballooning in rabbits [27]. These studies confirmed the previously shown role for GPVI in thrombosis, i.e. GPVI is required in the process of platelet recruitment under physiological shear stress in vivo. Therefore the feasibility of detecting thrombosis in its amplification stage was established in models mimicking vascular injury (wire-induced injury) or atherosclerotic plaque (ballooning). In addition, the GPVI strategy might detect also nascent thrombosis when it is triggered by collagen. Our findings add to these data with two important elements: first, we show the feasibility of detecting thrombosis generated by murine plaques, itself induced by hypercholesterolemia, as it is the case with human atherosclerosis. Second, our approach is different in regards to the molecular mechanism being targeted, because we used an antibody recognizing the final response of platelet activation and thus LIBS is able to recognize/detect atherothrombosis as soon as thrombosis is initiated, independent on the actual platelet stimulation pathway. Whether this difference is clinically relevant warrants further investigations.

Our results have some limitations that need to be considered. First, the scratching model does not provide “standardized” extent of atherothrombosis, since it depends mainly upon the plaque thrombogenicity, which is variable. In 7 of 20 cases animals had to be excluded from statistics because of a full occlusion led to a total blood signal void already in the baseline scans. Conversely, this variability in the atherothrombotic response is a good indication that this model is close to clinical pictures with plaque rupture and vessel occlusion.

Another limitation towards translation to human applications is the negative contrast generated by iron oxide based contrast agents. Iron oxides provide a reliable diagnosis only if native images express a hyper-intense signal which is altered by the contrast agent. However, ongoing research in MR methodology aiming for “white marker sequences” [28], [29] and the new technique of magnetic particle imaging [30] provide the potential to overcome these technical drawback. These new methodologies in combination with the LIBS targeting strategy should allow early detection of unstable atherosclerotic plaques in humans.

In conclusion, our studies provide the first proof of principle that atherothrombosis induced by murine plaque rupture can be detected by LIPS-MPIO. This detection was highly sensitive and constitutes therefore an important perspective for early detection of atherothrombosis in clinic and improvement in prevention of myocardial infarction.
